# Educational Effects of Ethnoscience, Indigenous Knowledge, and Local Wisdom in Science and STEM Education

**DOI:** 10.12688/f1000research.189428.1

**Published:** 2026-09-22

**Authors:** Lalu Usman Ali, Muhammad Fajar Marsuki, Sony Yunior Erlangga, Ratih Permana Sari, Nur Azizah Lubis, Florentina Maria Panda, Septi Aprilia, Dimas Fahrudin

**Affiliations:** 1 Doctoral Program in Natural Science Education, Universitas Sebelas Maret, Surakarta, Central Java, 57126, Indonesia; 2 Universitas Islam Negeri Mataram, Mataram, Nusa Tenggara Barat, 83116, Indonesia; 3 Natural Science Education, Universitas Negeri Malang, Malang, East Java, 65145, Indonesia; 4 University of Sarjanawiyata Tamansiswa, Yogyakarta, Special Region of Yogyakarta, 55167, Indonesia; 5 Samudra University, Langsa, Aceh, 24416, Indonesia; 6 Cenderawasih University, Jayapura, Papua, 99351, Indonesia; 7 Universitas Muhammadiyah PKU Surakarta, Surakarta, Central Java, 57136, Indonesia

**Keywords:** ethnoscience; Indigenous knowledge; local wisdom; science education; STEM education; culturally contextualized learning;

## Abstract

The integration of ethnoscience, Indigenous knowledge, local wisdom, and culturally contextualized pedagogy has been proposed as a means of improving the relevance of science and STEM education, yet the magnitude and consistency of its educational effects remain uncertain. This updated systematic review and meta-analysis synthesized 31 study-level estimates comprising 5,164 observations. The review followed PRISMA 2020, and intervention effects were expressed as bias-corrected standardized mean differences (Hedges’ g) and synthesized using an inverse-variance random-effects model with restricted maximum-likelihood estimation. Potential variation was examined across educational level, outcome type, instructional model, science subject, cultural context, research design, intervention duration, and media type. The pooled estimate favoured the intervention conditions (g = 1.17, 95% CI [0.88, 1.46], p < .001), but between-study heterogeneity was substantial (I ^2^ = 91.5%, τ ^2^ = 0.5766). Educational level, outcome type, instructional model, science subject, cultural context, and intervention duration did not show statistically detectable between-category differences. Media type produced a significant omnibus test, although several categories contained few studies and imprecise estimates, so this finding was treated as exploratory. The funnel plot showed possible asymmetry but did not establish publication bias. Overall, the evidence indicates a positive average association between culturally contextualized science interventions and educational outcomes, while the magnitude of effects varies considerably across studies. The findings support cautious, context-sensitive implementation and highlight the need for stronger designs, clearer intervention descriptions, and more consistent moderator coding.

## 1. Introduction

Science education is expected to help learners interpret natural phenomena, evaluate evidence, and participate in decisions involving science and technology. Yet formal science curricula are often presented through concepts, examples, and epistemic conventions that are detached from the cultural and material worlds in which students live. This separation can reduce perceived relevance and create discontinuities between learners’ everyday explanations and the discourse of school science. Aikenhead & Jegede, (1999) described this transition as cultural border crossing: students move between their life-worlds and the institutional culture of science, and the ease of that movement depends partly on how teaching acknowledges and mediates differences between knowledge systems ( Fan et al., 2025; Mund, 2025). From this perspective, improving science learning is not only a matter of simplifying scientific content; it also requires attention to the cultural resources through which learners interpret that content ( Erlangga et al., 2026; Fitriyah, Saputro, et al., 2026b; Kuncoro et al., 2026; Prihatni et al., 2026; Winingsih et al., 2023).

Ethnoscience, Indigenous knowledge, traditional ecological knowledge, and local wisdom refer to related but non-identical bodies of knowledge developed through long-term interaction with particular environments, communities, practices, and histories. In science education, these forms of knowledge can provide culturally meaningful phenomena, classifications, technologies, and explanatory practices through which disciplinary concepts are examined ( Juuso, 2024; Sakata et al., 2025). Snively & Corsiglia, (2001) argued that Indigenous sciences constitute systematic ways of knowing nature rather than collections of isolated traditions, while Zidny et al., (2020) emphasized their potential to broaden perspectives on nature, sustainability, and the relationship between humans and their environments. Their educational value, however, depends on respectful epistemic treatment. Cultural knowledge should not be reduced to decorative examples or positioned merely as raw material requiring validation by Western science ( Mallibhat, 2024; Mund, 2025). More defensible integration allows knowledge systems to be examined in relation to their purposes, evidence, contexts, limits, and ethical ownership ( Hsin, 2023; Tolbert et al., 2025).

The pedagogical rationale for culturally contextualized science learning is therefore multidimensional. First, familiar practices and local phenomena may activate prior knowledge and make abstract concepts more interpretable. Second, place-based and community-linked activities can provide authentic problems for inquiry, modelling, experimentation, and argumentation ( Stewart, 2021; Tolbert et al., 2025). Third, recognition of learners’ cultural resources may strengthen participation, identity, and perceived relevance. These mechanisms are compatible with constructivist and sociocultural accounts of learning, but they do not imply that cultural contextualization is sufficient by itself. The quality of teacher mediation, the accuracy of disciplinary connections, the depth of community involvement, and the alignment between activities and outcomes remain critical ( Ahanonye et al., 2026; Stewart, 2021). Culturally grounded instruction can be productive when it supports evidence-based comparison and dialogue; it can be superficial or misleading when cultural practices are decontextualized, romanticized, or treated as interchangeable with scientific explanations ( Ee et al., 2025; Le Marchand et al., 2025).

The empirical literature has expanded across countries, educational levels, scientific domains, and instructional formats. Sotero et al., (2020) documented a broad international literature linking local and scientific knowledge in school contexts and identified teacher education, community engagement, interdisciplinary activities, and culturally sensitive pedagogy as recurring features. A systematic review by Latip & Kadarohman, (2024) examined 52 studies published between 2014 and 2023 and reported increasing attention to local and Indigenous knowledge in science learning, including community- and place-based strategies and curriculum integration. Ogegbo & Ramnarain, (2024) likewise identified experiential activities, contextualized instructional materials, argumentative discussion, and engagement with knowledge keepers or elders as prominent pedagogical practices. Collectively, these reviews show that the field is no longer confined to a single instructional model or region; it encompasses inquiry, projects, collaborative learning, teacher professional development, digital media, simulations, modules, and curriculum-oriented approaches ( Ahanonye et al., 2026; Tolbert et al., 2025).

Despite this growth, the cumulative evidence remains difficult to interpret. Much of the review literature has focused on mapping concepts, practices, locations, and implementation challenges rather than estimating a pooled educational effect. Primary studies also differ in their populations, comparison conditions, outcome instruments, disciplinary content, intervention duration, and degree of cultural integration. Such variation can produce apparently inconsistent findings and makes it difficult to determine whether positive results are broadly distributed or concentrated in particular designs and contexts. A recent meta-analysis by Festiyed et al., (2025) synthesized 10 studies of ethnoscience-based culturally responsive science education among junior high school students and reported a positive average effect. That analysis provided useful quantitative evidence but was deliberately restricted to one educational stage and a relatively homogeneous set of studies. Questions therefore remain about the broader evidence base across primary, secondary, higher, and teacher education and across cognitive, affective, identity-related, and curriculum-oriented outcomes ( Boxerman, Sangueza, & Nelson-barber, 2026; Monk, 2023).

A broader synthesis is also needed because the label attached to an intervention may not identify the mechanism responsible for its effect. Ethnoscience may be delivered through inquiry, project-based STEM, collaborative experiential learning, digital tools, simulations, modules, games, or teacher-development programmes. Likewise, interventions may be coded as general context, local context, or Indigenous ethnoscience even though the depth of cultural integration varies substantially ( Peize & Sangiamvibool, 2025). Educational level, outcome domain, science subject, research design, duration, and media may therefore contribute to between-study variation. At the same time, moderator analyses in a heterogeneous literature require restraint: statistically nonsignificant differences cannot establish equivalence, and significant subgroup tests may be unstable when categories contain few studies or combine conceptually different interventions ( Amreta, 2024; Rathore et al., 2022; Wahyudi, 2023).

The present study responds to these limitations through an updated systematic review and random-effects meta-analysis of culturally contextualized science and STEM interventions. It extends prior syntheses by examining a broader range of educational levels, outcomes, instructional models, science subjects, cultural contexts, research designs, intervention durations, and media types ( Fianti & Neratania, 2024; Fitria, 2025). Hedges’ g was used to place outcomes measured on different scales onto a common metric, while heterogeneity and moderator analyses were used to evaluate the dispersion and possible conditionality of the effects. The purpose was not to determine whether cultural knowledge is universally superior to other forms of instruction, but to estimate the average association represented in the available quantitative evidence and to identify where that estimate is more or less stable. Accordingly, the study addressed three research questions: (1) What is the overall standardized effect associated with ethnoscience-, Indigenous knowledge-, local wisdom-, and culturally contextualized interventions in science and STEM education? (2) How much do the observed effects vary across studies? and (3) Do educational level, outcome type, instructional model, science subject, cultural context, research design, intervention duration, and media type account for statistically detectable variation in effect size?

## 2. Methods

### 2.1 Review design and reporting guideline

This study employed an updated systematic review and meta-analysis to synthesize quantitative evidence concerning the effects of ethnoscience, indigenous knowledge, local wisdom, and culturally contextualized instructional approaches in science and STEM education. An updated review design was adopted because the current synthesis incorporated one study retained from the previous version of the review and additional studies identified through an updated literature search. The review was conducted and reported in accordance with the Preferred Reporting Items for Systematic Reviews and Meta-Analyses 2020 guidelines ( Fitriyah et al., 2025; Fitriyah, Erlangga, et al., 2026b; Page et al., 2021). PRISMA 2020 was used to structure and document the identification, screening, eligibility assessment, and inclusion of studies, thereby supporting transparency, reproducibility, and completeness in the reporting process.

A review protocol was developed before the literature search and screening process. The protocol specified the review objectives, eligibility criteria, information sources, search strategy, study-selection procedures, data-extraction framework, moderator coding, risk-of-bias assessment, effect-size calculation, and statistical-analysis plan. The protocol was not formally registered in an external review registry. The review addressed three main objectives. First, it estimated the overall effect of ethnoscience- and culturally contextualized instructional interventions on science and STEM educational outcomes. Second, it evaluated the magnitude of between-study heterogeneity. Third, it examined whether variation in effect sizes was associated with educational level, outcome type, instructional model, science subject, cultural context, research design, intervention duration, and instructional media. The unit of analysis was the independent study-level comparison between an intervention group and a control or comparison group. Where multiple reports were derived from the same participants or dataset, the reports were linked and treated as a single underlying study to avoid double-counting participants.

### 2.2 Eligibility criteria

Studies were selected using predefined inclusion and exclusion criteria established before the screening process. These criteria were designed to ensure that the included studies were conceptually relevant to ethnoscience and science education and provided sufficient quantitative information for calculating standardized effect sizes. Eligible studies examined educational interventions that integrated ethnoscience, indigenous knowledge, indigenous science, local wisdom, traditional ecological knowledge, culturally grounded science, culture-based learning, culturally responsive science education, or ethno-STEM into science or STEM instruction. Participants could include primary school students, secondary school students, higher education students, pre-service teachers, or in-service teachers involved in science- or STEM-related education.

The quantitative synthesis included studies employing experimental, quasi-experimental, or controlled comparative designs. Studies were required to provide sufficient statistical information to calculate a standardized mean difference, including sample sizes, means, standard deviations, standard errors, confidence intervals, or other convertible statistics. Eligible educational outcomes included academic achievement, scientific knowledge, conceptual understanding, scientific literacy, thinking and reasoning skills, motivation, awareness, attitudes, self-efficacy, and science-related identity. Studies were excluded when they were conceptual, qualitative, descriptive, correlational, non-intervention, or review studies, or when they did not provide sufficient quantitative information for effect-size calculation. The detailed inclusion and exclusion criteria are presented in Table 1.

**Table 1.** Inclusion and exclusion criteria.

Category	Inclusion criteria	Exclusion criteria
***Research focus***	*Examined ethnoscience, indigenous knowledge, local wisdom, indigenous science, culturally grounded science, culture-based learning, or ethno-STEM in science or STEM education*	*Examined topics unrelated to ethnoscience, cultural knowledge, science education, or STEM education*
***Participants***	*Included primary, secondary, or higher education students, pre-service teachers, or in-service teachers involved in science or STEM education*	*Included participants outside an educational context or samples unrelated to science or STEM education*
***Research design***	*Used an experimental, quasi-experimental, or controlled comparative design involving an intervention and a comparison condition*	*Used a conceptual, qualitative, descriptive, correlational, review, or non-intervention design*
***Intervention***	*Implemented a structured instructional approach incorporating ethnoscience, local knowledge, indigenous knowledge, or culturally contextualized science content*	*Used general or conventional instruction without an identifiable cultural, indigenous, local-knowledge, or ethnoscientific component*
***Comparator***	*Included conventional instruction, standard curriculum, an alternative instructional approach, or another clearly defined control or comparison condition*	*Did not provide a valid comparison condition or presented data that prevented comparison between intervention and control groups*
***Learning outcomes***	*Reported achievement, knowledge, conceptual understanding, scientific literacy, reasoning skills, thinking skills, motivation, attitudes, awareness, self-efficacy, or science-related identity*	*Reported outcomes unrelated to science or STEM education or provided only descriptive perceptions without measurable educational outcomes*
***Data availability***	*Reported sample size, mean, and standard deviation for the intervention and comparison groups or provided convertible quantitative statistics*	*Did not provide sufficient statistical information to calculate Hedges’ g*
***Subject area***	*Focused on biology, environmental science, physics, earth science, chemistry, integrated science, or general STEM education*	*Focused exclusively on non-science subjects without a direct science or STEM component*
***Publication type***	*Full-text peer-reviewed journal article*	*Thesis, dissertation, conference abstract, editorial, review article, book chapter, report, or unpublished manuscript*
***Language***	*Published in English*	*Published in another language without an accessible and reliable English version*
***Publication status***	*Final published article with accessible methodological and statistical information*	*Retracted, incomplete, inaccessible, or insufficiently documented publication*
***Duplicate data***	*Represented an independent sample, intervention, or dataset*	*Duplicated participants, data, or results already reported in another included publication*

When multiple publications reported findings from the same participants, intervention, or dataset, they were treated as a single study. The publication containing the most complete methodological description and quantitative data was retained. This procedure was used to maintain the statistical independence of the included effect sizes and prevent the same participants from being counted more than once.

### 2.3 Information sources and search strategy

A comprehensive literature search was conducted across international bibliographic databases, research registers, relevant websites, organisational sources, and citation networks. Database and register searching was supplemented by backward reference-list screening, forward citation searching, targeted website searching, and searches of relevant educational and research organisations. The search strategy consisted of five conceptual components: cultural knowledge, science or STEM education, educational participants, intervention research, and educational outcomes. Terms related to cultural knowledge included “ethnoscience,” “indigenous knowledge,” “indigenous science,” “local wisdom,” “traditional ecological knowledge,” “culturally grounded science,” “culturally responsive science,” “culture-based science,” and “ethno-STEM.”

These terms were combined with science-education terms, including “science education,” “science learning,” “STEM education,” “biology education,” “physics education,” “chemistry education,” “earth science,” and “environmental science.” Participant-related terms included “student,” “learner,” “pupil,” “teacher,” “pre-service teacher,” and “in-service teacher.” Research-design terms included “experiment,” “intervention,” “quasi-experiment,” “control group,” “comparison group,” “pretest,” and “posttest.” Outcome-related terms included “achievement,” “knowledge,” “conceptual understanding,” “scientific literacy,” “reasoning,” “thinking skills,” “motivation,” “attitude,” “awareness,” “self-efficacy,” and “identity.”

The general search structure was as follows:

(“ethnoscience” OR “indigenous knowledge” OR “indigenous science” OR “local wisdom” OR “traditional ecological knowledge” OR “culturally grounded science” OR “culturally responsive science” OR “culture-based science” OR “ethno-STEM”) AND (“science education” OR “science learning” OR “STEM education” OR biology OR physics OR chemistry OR “earth science” OR “environmental science”) AND (student* OR learner* OR pupil* OR teacher* OR “pre-service teacher*” OR “in-service teacher*”) AND (experiment* OR intervention* OR quasi-experiment* OR “control group” OR “comparison group” OR pretest OR posttest) AND (achievement OR knowledge OR “conceptual understanding” OR “scientific literacy” OR reasoning OR “thinking skills” OR motivation OR attitude* OR awareness OR self-efficacy OR identity)

The search syntax was adapted to the field codes, phrase-searching functions, truncation rules, and Boolean requirements of each information source. Searches were applied to titles, abstracts, and keywords whenever these fields were available. No restriction based on educational level was applied during the initial search. Broad search terms were used to maximize search sensitivity, while the relevance and eligibility of the retrieved publications were determined during title and abstract screening and full-text assessment. Reference lists of potentially eligible studies were examined to identify additional publications ( Arztmann, 2023; Budniok et al., 2026). Forward citation searches were also conducted to locate more recent studies that cited relevant publications. All records retrieved from the different sources were exported to reference-management software before deduplication and screening. The search identified 3,360 records from bibliographic databases and 37 records from research registers. An additional 100 records were identified through supplementary methods, comprising 42 records from websites, 18 from organisations, 25 through citation searching, and 15 from other relevant sources.

### 2.4 Study selection process

The study-selection process followed the identification, screening, eligibility, and inclusion stages specified in PRISMA 2020. Records identified through databases, registers, websites, organisations, citation searching, and other supplementary sources were compiled into a single review database using reference-management software. Duplicate records were initially identified through automated matching of titles, authors, publication years, and digital identifiers. The automatically detected duplicates were subsequently checked manually to prevent the incorrect removal of publications with similar titles or authorship information ( Ding, 2023; Zohar & Ben-Ari, 2022).

Before title and abstract screening, 412 duplicate records were removed. An automated screening procedure marked 150 records as ineligible based on predefined bibliographic and content-related criteria. A further 50 records were removed for other predefined reasons, including clearly ineligible publication types, incomplete records, and documents outside the scope of the review. Following these removals, 2,785 records remained for title and abstract screening. Title and abstract screening was conducted independently by two reviewers using systematic-review screening software. Each record was assessed against the predefined eligibility criteria. Records were excluded at this stage when they clearly failed to address ethnoscience, indigenous knowledge, local wisdom, culturally contextualized instruction, science education, STEM education, relevant participant groups, educational interventions, or measurable educational outcomes.

The reviewers completed the screening independently before comparing their decisions. Disagreements were resolved through discussion and consensus. When agreement could not be reached, a third reviewer adjudicated the eligibility decision. Of the 2,785 records screened, 2,618 were excluded during title and abstract screening. The remaining 167 reports were sought for full-text retrieval. Seven reports could not be retrieved because the full text was unavailable, resulting in 160 reports assessed for eligibility. Full-text assessment was also conducted independently by two reviewers. Each report was evaluated against all inclusion and exclusion criteria presented in Table 1. The reviewers recorded one primary reason for excluding each ineligible report to avoid double-counting exclusion reasons.

Among the 160 reports assessed from the database and register pathway, 133 were excluded. The reasons were an irrelevant topic in 58 reports, an ineligible research design in 39 reports, insufficient quantitative data in 25 reports, and other eligibility-related reasons in 11 reports. The supplementary search pathway identified 100 reports. Five reports could not be retrieved, leaving 95 reports for full-text eligibility assessment. Of these, 92 were excluded because 40 were off-topic, 27 employed an ineligible research design, 18 lacked sufficient quantitative information, and seven did not meet other eligibility criteria. The updated search produced 30 newly eligible studies. One study was retained from the previous version of the review, resulting in a total of 31 studies included in the systematic review and meta-analysis. The complete study-selection process is presented in the PRISMA 2020 flow diagram in Figure 1.

**Figure 1.**
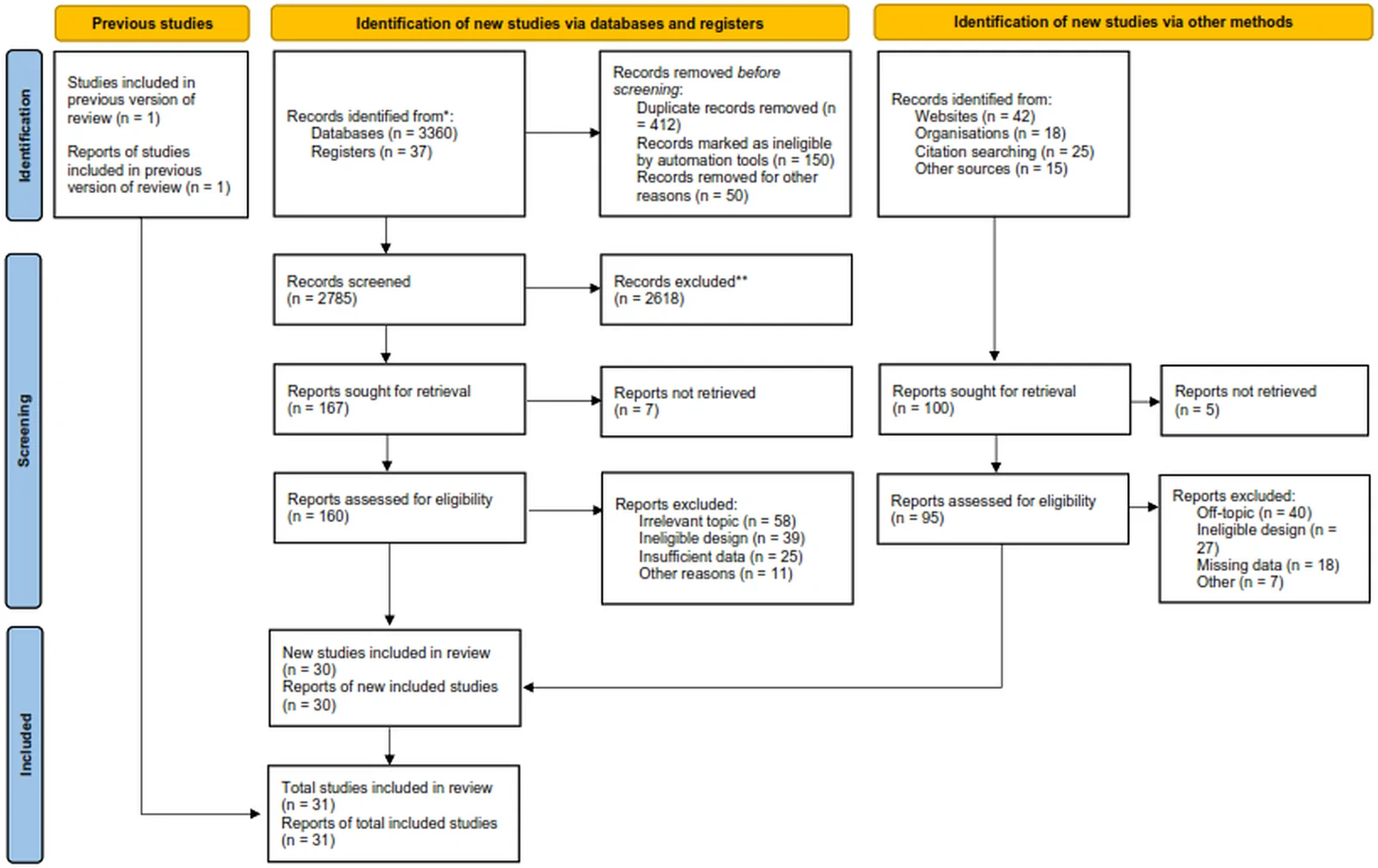
PRISMA 2020 flow diagram of study identification, screening, eligibility assessment, and inclusion.

### 2.5 Data extraction and moderator coding

A standardized data-extraction form was used to record study identification, publication year, participant characteristics, educational level, research design, intervention characteristics, outcome measures, sample sizes, means, and standard deviations. When necessary, other reported statistics were converted into a format suitable for effect-size calculation. Data were extracted from the main text, tables, figures, and supplementary materials and were independently verified by a second reviewer. Discrepancies were resolved by rechecking the original publication. Eight moderators were coded to examine potential sources of variation in effect sizes: educational level, outcome type, instructional model, science subject, cultural context, research design, intervention duration, and media type. These moderators were selected because participant characteristics, pedagogical approaches, disciplinary content, cultural integration, methodological design, intervention intensity, and learning resources may influence intervention outcomes. The operational definitions and coding categories for these moderator variables are summarized in Table 2.

**Table 2.** Coding framework for moderator variables.

Moderator	Operational definition	Coding categories
***Education Level***	*Educational level of participants receiving the intervention*	*Primary; Secondary; Higher/Teacher Education*
***Outcome Type***	*Main educational outcome measured in the study*	*Achievement and Knowledge; Thinking and Reasoning Skills; Conceptual Literacy; Motivation, Awareness, and Attitudes; Self-Efficacy and Identity*
***Instructional Model***	*Main pedagogical approach used in the intervention*	*Media-Assisted Learning; Collaborative Experiential Learning; Inquiry/Project-Based STEM; Culture-Context-Based Learning*
***Science Subject***	*Main disciplinary area addressed in the intervention*	*Biology and Environment; Physics and Earth Science; General STEM*
***Cultural Context***	*Type of cultural integration represented in the intervention*	*General Context; Local Context; Indigenous Ethnoscience*
***Research Design***	*Methodological structure used to estimate the intervention effect*	*Pretest–Posttest Controlled; Pretest–Posttest One-Group; Pretest–Posttest Mixed Methods*
***Intervention Duration***	*Length of intervention implementation*	*Short; Medium; Long*
***Media Type***	*Main instructional medium or learning resource*	*AI and Digital Tools; Simulation and Animation; Module and Activity Materials; Digital Platform and Open Educational Resources; Game and Narrative Media*

Coding was based on the primary participant group, principal outcome, dominant instructional model, main science subject, central cultural orientation, and main instructional medium reported in each study. Duplicate reports, conceptual studies, and studies unsuitable for quantitative synthesis were not treated as moderator categories.

### 2.6 Methodological quality and risk-of-bias assessment

The methodological quality of the included studies was assessed using a structured risk-of-bias framework appropriate for experimental and quasi-experimental educational research. The assessment covered participant selection, baseline comparability, group allocation, intervention implementation, missing outcome data, outcome measurement, and selective reporting. Each domain was rated as low risk, some concerns, or high risk of bias. An overall judgment was assigned based on the pattern and severity of domain-level concerns. Two reviewers independently completed the assessment, and disagreements were resolved through discussion or consultation with a third reviewer. Risk-of-bias judgments were not converted into numerical quality scores and were not used as statistical weights. Instead, they were considered in the interpretation of findings and in sensitivity analyses examining whether the pooled estimate changed after studies with high risk of bias were excluded.

### 2.7 Effect-size calculation and meta-analytic model

The intervention effect was expressed as Hedges’ *g*, a bias-corrected standardized mean difference. This measure was used because the included studies assessed related educational outcomes using different instruments and scoring scales. Positive effect sizes indicated more favourable outcomes for the intervention group. For outcomes in which lower scores represented better performance, the effect direction was reversed. The quantitative synthesis included 31 independent study-level effects and 5,164 observations, consisting of 2,580 observations in intervention groups and 2,584 in comparison groups. Each study contributed one independent effect estimate to the primary model. When a study reported multiple relevant outcomes, the primary outcome was selected or closely related measures were combined to avoid overrepresentation of a single study. Reports based on the same sample or dataset were treated as one independent study ( Gunnoo et al., 2023; Ribeiro et al., 2026).

A random-effects model was applied because the true intervention effects were expected to vary across educational levels, instructional models, science subjects, cultural contexts, research designs, intervention durations, media types, and outcome measures. Studies were weighted using the inverse-variance method, while between-study variance was estimated using restricted maximum likelihood. Confidence intervals for the heterogeneity variance were calculated using the Q-profile method. All effect estimates were reported with 95% confidence intervals, and statistical significance was set at *p* < .05.

### 2.8 Heterogeneity and moderator analysis

Between-study heterogeneity was assessed using Cochran’s Q, I ^2^, τ ^2^, τ, and H. These statistics were interpreted jointly with the magnitude, direction, and precision of individual effect sizes. Substantial heterogeneity was considered evidence that study-level characteristics could contribute to variation in intervention effects. Potential sources of heterogeneity were examined using subgroup analyses and mixed-effects meta-regression ( Maringa et al., 2025; Varah et al., 2025). The prespecified moderators were educational level, outcome type, instructional model, science subject, cultural context, research design, intervention duration, and media type. Each moderator was first examined separately using random-effects subgroup analysis or univariable meta-regression. Differences between moderator categories were evaluated using an omnibus test rather than by comparing the significance of individual subgroup estimates. Categories represented by too few studies were combined when conceptually appropriate or reported descriptively.

Because the meta-analysis included 31 studies, multivariable meta-regression was restricted to a limited number of theoretically relevant predictors to reduce the risk of overfitting. Educational level, outcome type, instructional model, cultural context, and intervention duration were treated as the principal substantive moderators. Science subject and media type were examined as additional instructional moderators, while research design was treated as a methodological moderator and was also considered in sensitivity analysis. Moderator findings were interpreted cautiously because subgroup sizes were unequal and several study characteristics could be correlated. Interpretation therefore considered effect magnitude, confidence intervals, residual heterogeneity, theoretical plausibility, and the distribution of studies across moderator categories.

### 2.9 Statistical software

All statistical analyses were conducted using R within the RStudio integrated development environment. The meta package was used to calculate Hedges’ *g*, estimate the pooled effect under the random-effects model, assess heterogeneity, conduct subgroup analyses, and generate forest and funnel plots. The metafor package was used for meta-regression, influence diagnostics, and sensitivity analyses. Studies were weighted using the inverse-variance method, and between-study variance was estimated using the restricted maximum-likelihood estimator ( Wood & Hedges, 2024). Confidence intervals for τ ^2^ were calculated using the Q-profile method. Statistical tests were two-sided, with p < .05 considered statistically significant. All data-processing procedures, coding decisions, and statistical outputs were checked before interpretation to ensure consistency between the analytical dataset and the reported results.

## 3. Results

### 3.1 Study selection

The study-selection process is presented in Figure 1. The search identified 3,497 records, comprising 3,360 records from bibliographic databases, 37 from research registers, and 100 from supplementary sources. The supplementary records consisted of 42 records identified through websites, 18 through organisations, 25 through citation searching, and 15 through other sources. Before screening, 412 duplicate records were removed, 150 records were marked as ineligible through the automated screening procedure, and 50 records were removed for other predefined reasons. This process left 2,785 records for title and abstract screening, of which 2,618 were excluded because they did not meet the preliminary eligibility criteria.

From the database and register pathway, 167 reports were sought for retrieval. Seven reports could not be retrieved, leaving 160 reports for full-text eligibility assessment. Following full-text review, 133 reports were excluded because they addressed an irrelevant topic (n = 58), employed an ineligible research design (n = 39), lacked sufficient quantitative information (n = 25), or did not meet other eligibility requirements (n = 11). From the supplementary-search pathway, 100 reports were sought for retrieval. Five reports could not be obtained, and 95 were assessed in full text. Of these, 92 were excluded because they were off-topic (n = 40), used an ineligible research design (n = 27), lacked sufficient quantitative data (n = 18), or failed to meet other eligibility criteria (n = 7). The updated search yielded 30 newly eligible studies. Together with one study retained from the previous version of the review, 31 studies were included in the final systematic review and quantitative synthesis.

### 3.2 Overall effect and between-study heterogeneity

The quantitative synthesis included 31 study-level estimates and 5,164 observations, consisting of 2,580 observations in the intervention conditions and 2,584 in the comparison conditions. As shown in Figure 2, the random-effects model produced a positive pooled standardized mean difference of g = 1.17, 95% CI [0.88, 1.46], z = 7.83, p < .001. The average standardized difference therefore favoured the intervention conditions. Nevertheless, the pooled estimate represents an average across studies with substantially different effect sizes and should not be interpreted as a uniform effect across all settings. The individual effect estimates displayed in Figure 2 ranged from −0.03 to 4.74.

**Figure 2.**
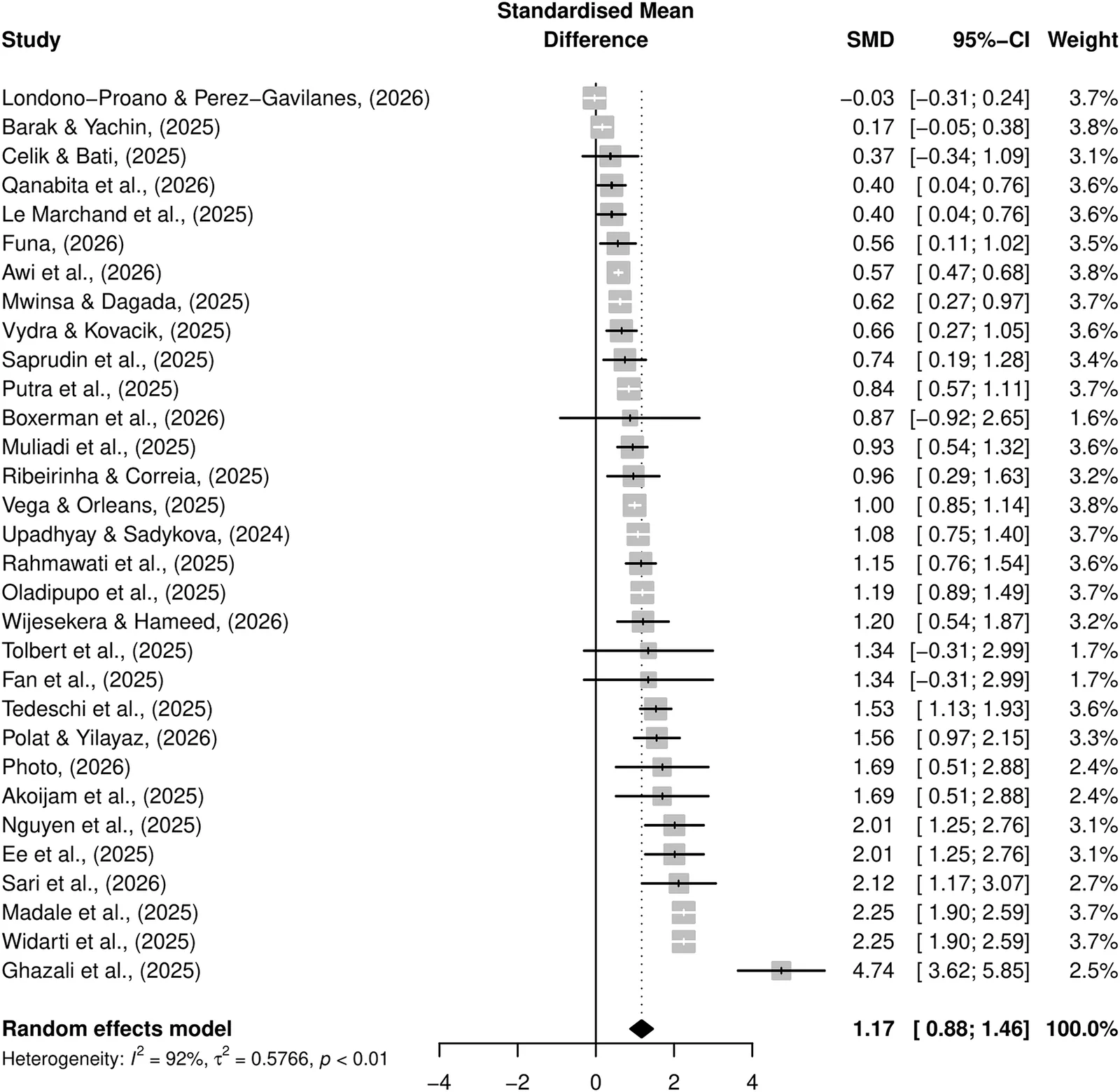
Forest plot of individual and pooled standardized mean differences under the random-effects model.

Several estimates had confidence intervals that included zero, indicating considerable statistical uncertainty at the individual-study level. For example, the estimate for Londoño-Proaño and Pérez-Gavilanes was close to zero, g = −0.03, 95% CI [−0.31, 0.24], whereas the largest estimate was reported for Ghazali et al., g = 4.74, 95% CI [3.62, 5.85]. Most plotted estimates were positive, but their magnitude and precision varied considerably. Substantial between-study heterogeneity was observed, Q(30) = 354.11, p < .001, I ^2^ = 91.5%, τ ^2^ = 0.5766, τ = 0.7593, and H = 3.44. Figure 2 reports I ^2^ in rounded form as 92%. These statistics indicate that the variability among the observed effect estimates was considerably greater than would be expected from sampling error alone. The high heterogeneity suggests that the magnitude of the standardized differences may vary according to study population, instructional approach, outcome construct, disciplinary context, research design, intervention duration, media characteristics, or other unmeasured study-level factors. Consequently, the pooled result was interpreted alongside the moderator analyses rather than as an effect expected to occur consistently in every educational context.

### 3.3 Moderator analysis

Moderator analyses were conducted to examine whether variation in effect estimates was associated with educational level, outcome type, instructional model, science subject, cultural context, media type, and intervention duration. Figure 3 presents the number of studies, subgroup-specific effect estimates, confidence intervals, and omnibus interaction tests for each moderator, while Table 3 summarizes the corresponding moderator results and their statistical interpretations.

**Figure 3.**
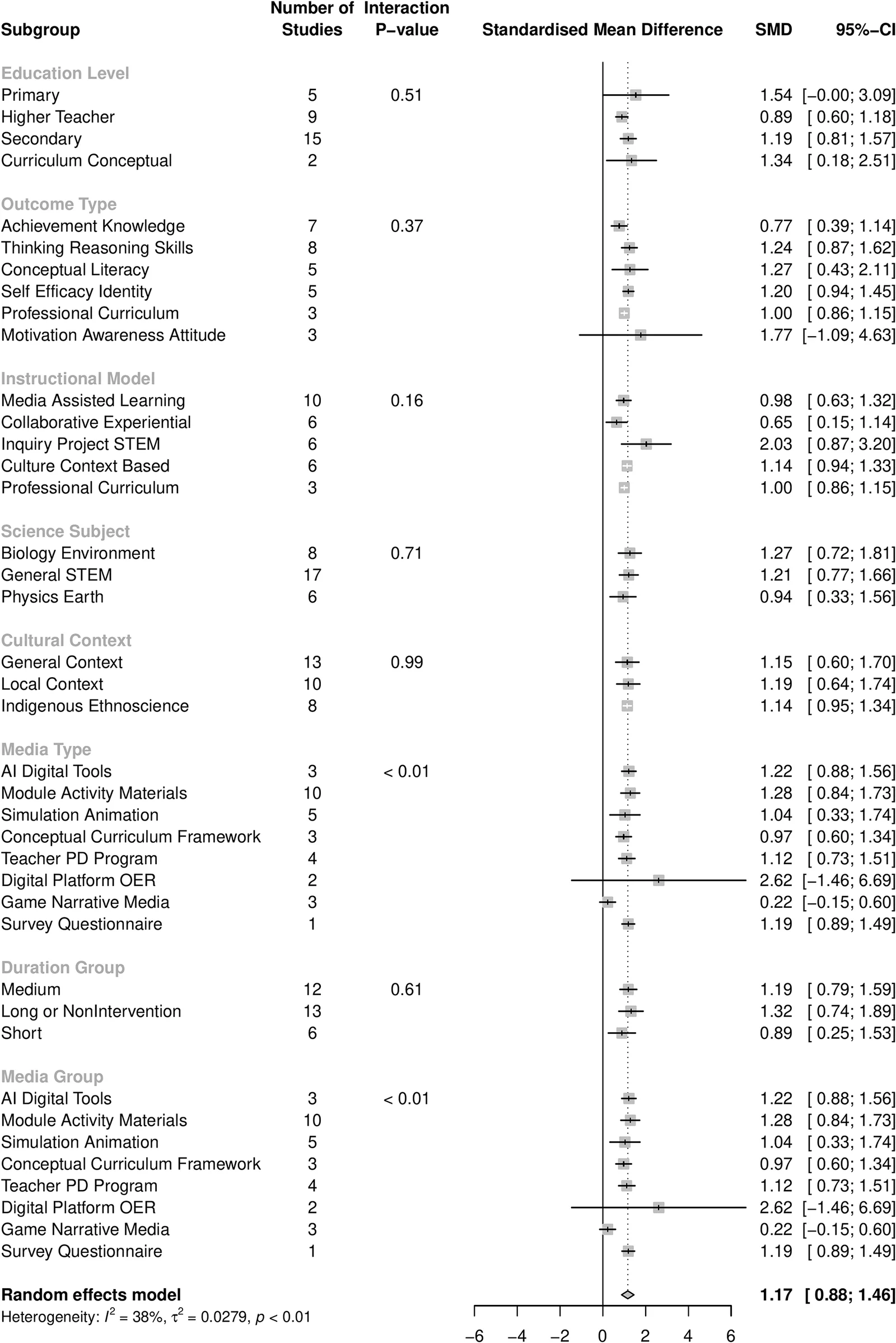
Subgroup analyses according to educational level, outcome type, instructional model, science subject, cultural context, media type, and intervention duration.

**Table 3.** Summary of moderator analyses.

Moderator	Range of subgroup estimates	Interaction p-value	Statistical interpretation
***Education level***	*0.89–1.54*	*.51*	*No statistically detectable difference*
***Outcome type***	*0.77–1.77*	*.37*	*No statistically detectable difference*
***Instructional model***	*0.65–2.03*	*.16*	*No statistically detectable difference*
***Science subject***	*0.94–1.27*	*.71*	*No statistically detectable difference*
***Cultural context***	*1.14–1.19*	*.99*	*No statistically detectable difference*
***Media type***	*0.22–2.62*	*< .01*	*Statistically detectable between-category variation*
***Intervention duration***	*0.89–1.32*	*.61*	*No statistically detectable difference*

**3.3.1 Education level**

The subgroup analysis included five estimates classified as primary education, nine as higher or teacher education, 15 as secondary education, and two as curriculum-conceptual studies. The pooled estimates were g = 1.54, 95% CI [−0.00, 3.09], for primary education; g = 0.89, 95% CI [0.60, 1.18], for higher or teacher education; g = 1.19, 95% CI [0.81, 1.57], for secondary education; and g = 1.34, 95% CI [0.18, 2.51], for curriculum-conceptual studies. The omnibus interaction test was not statistically significant, p = .51. Thus, although the subgroup point estimates differed numerically, the analysis did not provide sufficient evidence that effect magnitude varied systematically across educational levels. The primary-education estimate was also imprecise, as indicated by its wide confidence interval.

**3.3.2 Outcome type**

The pooled standardized mean differences were g = 0.77, 95% CI [0.39, 1.14], for achievement and knowledge; g = 1.24, 95% CI [0.87, 1.62], for thinking and reasoning skills; g = 1.27, 95% CI [0.43, 2.11], for conceptual literacy; g = 1.20, 95% CI [0.94, 1.45], for self-efficacy and identity; g = 1.00, 95% CI [0.86, 1.15], for professional curriculum outcomes; and g = 1.77, 95% CI [−1.09, 4.63], for motivation, awareness, and attitudes. The omnibus interaction test was not statistically significant, p = .37. The available data therefore did not indicate statistically detectable differences among outcome categories. The motivation, awareness, and attitudes subgroup showed the largest point estimate, but its wide confidence interval indicates substantial uncertainty.

**3.3.3 Instructional model**

The pooled estimates were g = 0.98, 95% CI [0.63, 1.32], for media-assisted learning; g = 0.65, 95% CI [0.15, 1.14], for collaborative experiential learning; g = 2.03, 95% CI [0.87, 3.20], for inquiry- or project-based STEM; g = 1.14, 95% CI [0.94, 1.33], for culture-context-based learning; and g = 1.00, 95% CI [0.86, 1.15], for professional curriculum approaches. Although inquiry- or project-based STEM had the largest subgroup point estimate, the omnibus interaction test was not statistically significant, p = .16. Accordingly, the observed numerical differences should not be interpreted as evidence that one instructional model was consistently more effective than the others.

**3.3.4 Science subject**

The subgroup estimates were g = 1.27, 95% CI [0.72, 1.81], for biology and environmental science; g = 1.21, 95% CI [0.77, 1.66], for general STEM; and g = 0.94, 95% CI [0.33, 1.56], for physics and earth science. The omnibus interaction test was not statistically significant, p = .71. The overlapping confidence intervals and nonsignificant test indicate that the analysis did not detect systematic variation in effect magnitude across science-subject categories.

**3.3.5 Cultural context**

The estimated effects were highly similar across cultural-context categories. General-context studies produced g = 1.15, 95% CI [0.60, 1.70]; local-context studies produced g = 1.19, 95% CI [0.64, 1.74]; and studies coded as indigenous ethnoscience produced g = 1.14, 95% CI [0.95, 1.34]. The omnibus interaction test was not statistically significant, p = .99. The current analysis therefore did not indicate that effect magnitude differed according to the cultural-context classification used in the dataset.

**3.3.6 Media type**

The media-type estimates were g = 1.22, 95% CI [0.88, 1.56], for AI and digital tools; g = 1.28, 95% CI [0.84, 1.73], for modules and activity materials; g = 1.04, 95% CI [0.33, 1.74], for simulations and animations; g = 0.97, 95% CI [0.60, 1.34], for conceptual curriculum frameworks; g = 1.12, 95% CI [0.73, 1.51], for teacher professional-development programmes; g = 2.62, 95% CI [−1.46, 6.69], for digital platforms and open educational resources; g = 0.22, 95% CI [−0.15, 0.60], for game- and narrative-based media; and g = 1.19, 95% CI [0.89, 1.49], for the survey-questionnaire category. The omnibus interaction test was statistically significant, p < .01, suggesting that effect estimates differed across the media categories as coded in the dataset. However, this result should be interpreted cautiously. Several subgroups contained only one to three estimates, and the digital-platform and open-educational-resource subgroup had a particularly wide confidence interval. The statistically significant omnibus test therefore indicates possible between-category variation but does not establish that a particular medium is consistently superior.

**3.3.7 Intervention duration**

The estimated effects were g = 1.19, 95% CI [0.79, 1.59], for medium-duration interventions; g = 1.32, 95% CI [0.74, 1.89], for the long-or-non-intervention category; and g = 0.89, 95% CI [0.25, 1.53], for short interventions. The omnibus interaction test was not statistically significant, p = .61. Thus, the analysis did not detect systematic differences across the duration categories. Nevertheless, the “long or non-intervention” category combines conceptually different conditions, so its estimate should be treated as provisional until long-duration interventions and non-intervention studies are coded separately. Overall, media type was the only moderator with a statistically significant omnibus interaction test. The remaining moderators showed numerical variation in subgroup point estimates, but their interaction tests were not statistically significant. These findings should be treated as exploratory because several subgroups were small, confidence intervals were sometimes wide, and some coding categories combined methodologically different study characteristics.

### 3.4 Funnel plot and potential small-study effects

The funnel plot is presented in Figure 4. Most studies were concentrated around standardized mean differences between approximately 0 and 2, with standard errors generally below 0.40. However, several studies were positioned outside the central region of the funnel, including one study with a standardized mean difference close to 4.7 and several studies with relatively large standard errors. The distribution was therefore not fully symmetrical. Visual asymmetry may indicate potential small-study effects, selective reporting, or differences in the methodological characteristics of smaller and larger studies. Nevertheless, the funnel plot alone does not provide sufficient evidence of publication bias. The observed pattern may also reflect substantial between-study heterogeneity, variation in research design, differences in outcome measurement, or differences in intervention and participant characteristics. Because the meta-analysis showed high heterogeneity, the funnel plot should be interpreted cautiously. A formal statistical test of funnel-plot asymmetry, such as Egger’s regression test, would be required to determine whether the asymmetry was statistically detectable. In the absence of a formal test result, the current evidence suggests possible asymmetry but does not confirm publication bias.

**Figure 4.**
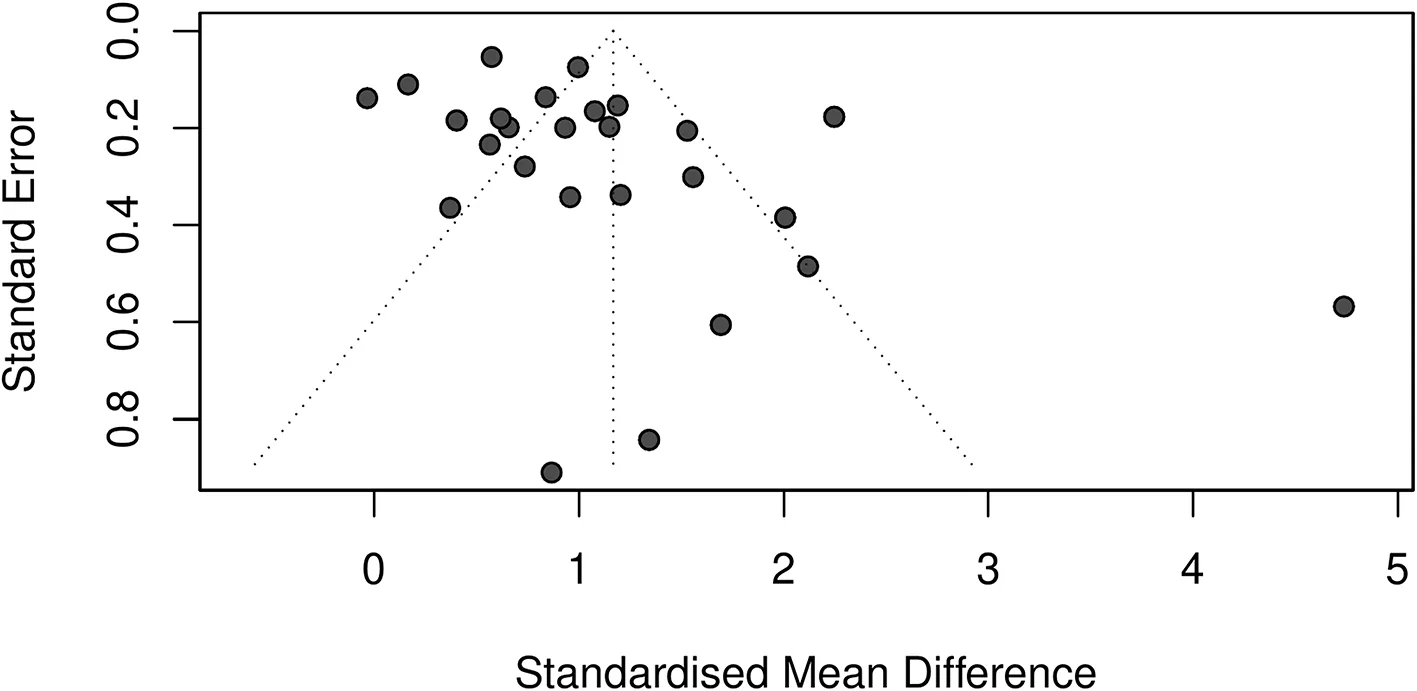
Funnel plot of standardized mean differences against standard errors.

### 3.5 Summary of the main findings

The random-effects meta-analysis of 31 study-level estimates produced a pooled standardized mean difference of g = 1.17, 95% CI [0.88, 1.46], indicating an average difference in favour of the intervention conditions. However, the effect estimates varied considerably across studies, ranging from approximately −0.03 to 4.74. The high heterogeneity, I ^2^ = 91.5%, indicates that the pooled effect should be interpreted as the mean of a broad distribution of study effects rather than as a uniform effect across educational contexts.

The moderator analyses did not identify statistically detectable differences according to educational level, outcome type, instructional model, science subject, cultural context, or intervention duration. Media type was the only moderator with a statistically significant omnibus interaction test. Nevertheless, this finding should be regarded as exploratory because several media categories contained few studies and some subgroup estimates had wide confidence intervals. The funnel plot showed possible asymmetry, but this pattern cannot be attributed exclusively to publication bias. Substantial heterogeneity and differences in study design, sample size, outcome measurement, and intervention characteristics may also have contributed to the observed distribution. Accordingly, the results indicate a positive average association between the examined interventions and educational outcomes, while the magnitude and consistency of the effects varied across the included studies ( Gulnara et al., 2025).

## 4. Discussion

The present meta-analysis identified a positive average association between the examined ethnoscience-, indigenous knowledge-, local wisdom-, and culturally contextualized interventions and educational outcomes in science and STEM. Across 31 study-level estimates and 5,164 observations, the random-effects model yielded a pooled standardized mean difference of g = 1.17, 95% CI [0.88, 1.46]. In standardized terms, this estimate is comparatively large; however, it should not be interpreted as evidence that every intervention produced an effect of this magnitude. The very high heterogeneity (I ^2^ = 91.5%) and the broad range of individual effects, from −0.03 to 4.74, indicate that the pooled value represents the average of substantially different intervention effects. The main substantive conclusion is therefore not that ethnoscience-based instruction is uniformly effective, but that the available evidence tends to favour contextualized intervention conditions while showing considerable variation in magnitude, precision, and likely transferability across settings.

The positive overall pattern is theoretically consistent with cross-cultural perspectives on science learning. Students encounter formal science through prior knowledge, language, values, practices, and explanatory systems developed in their families and communities. Aikenhead and Jegede (1999) conceptualized movement between everyday lifeworlds and school science as a form of cultural border crossing, with learning shaped by how students negotiate similarities and tensions between knowledge systems. From this perspective, ethnoscience and culturally grounded instruction may support learning when they make scientific ideas more interpretable, connect abstract concepts to familiar phenomena, and create opportunities for students to compare explanations using evidence. This mechanism is more defensible than assuming that the mere inclusion of cultural content automatically improves achievement. The instructional value of cultural knowledge depends on how it is selected, represented, discussed, and connected to scientific inquiry.

The pooled estimate may also reflect the combined influence of contextual relevance and active pedagogy. Many of the included interventions were not limited to presenting local or Indigenous examples; they also incorporated inquiry, projects, collaboration, simulations, modules, or digital resources. These features can increase opportunities for observation, questioning, explanation, and application. Hsin, (2023), for example, reported improvement in young children’s scientific practices following a structured project-based science module implemented in urban and Indigenous settings, emphasizing the role of intentional guidance and scaffolding. This suggests that the benefits attributed to ethnoscience may partly arise from the interaction between meaningful context and well-structured pedagogy. The present synthesis cannot isolate these mechanisms completely because cultural content, instructional model, media, and duration often co-occurred within the same studies ( Ahmmad et al., 2025; Amreta, 2024).

The high heterogeneity is therefore a central finding rather than a secondary statistical detail. Several factors may have contributed to the dispersion of effects, including differences in participant age, teacher expertise, disciplinary content, comparison conditions, intervention intensity, outcome instruments, and methodological quality. The largest study-level effect, g = 4.74, is unusually high for an educational intervention and may exert disproportionate influence on the pooled estimate. Such an effect does not necessarily indicate an error, but it warrants examination of sample size, baseline comparability, instrument variability, and whether the outcome measure was closely aligned with the intervention. Conversely, several confidence intervals crossed zero, showing that positive effects were not statistically distinguishable in every study. A prediction interval would strengthen the interpretation by indicating the range of true effects that might plausibly occur in a comparable future setting, because a confidence interval around the pooled mean does not communicate the full dispersion of random effects ( IntHout et al., 2016).

The moderator analyses provided limited evidence that the average effect differed systematically across educational levels or outcome domains. Although the subgroup point estimates varied, neither educational level nor outcome type produced a statistically significant omnibus test. This does not demonstrate equivalence among primary, secondary, and higher or teacher education, nor among cognitive and affective outcomes. Moderator tests often have limited power when the number of studies is modest, subgroup sizes are unequal, and within-subgroup heterogeneity remains substantial. The wide confidence interval for primary education and the particularly imprecise estimate for motivation, awareness, and attitudes illustrate this problem ( Hsu, 2022; Prayogi et al., 2023). The current results are therefore more appropriately interpreted as an absence of statistically detectable differences than as evidence that age, educational stage, or outcome type is irrelevant ( Putra et al., 2025; Rahmawati et al., 2025).

A similar caution applies to instructional model and science subject. Inquiry- and project-based STEM produced the largest point estimate among instructional models, but the between-model test was not statistically significant. The estimate is theoretically plausible because inquiry and project structures can position cultural phenomena as objects of investigation rather than as supplementary examples. Nevertheless, the data do not establish that inquiry- or project-based STEM is superior to media-assisted, collaborative experiential, or culture-context-based instruction. Instructional model may also be confounded with intervention duration, media use, outcome choice, and teacher support. Likewise, the nonsignificant differences among biology and environmental science, general STEM, and physics and earth science suggest that contextualized approaches may be adaptable across domains, but the broad subject categories may conceal important disciplinary variation ( Amreta, 2024; Muliadi et al., 2025). General STEM, in particular, combines interventions with potentially different epistemic and curricular demands.

The cultural-context analysis requires especially careful interpretation. The estimates for general context, local context, and Indigenous ethnoscience were almost identical, and the omnibus test was nonsignificant. This finding does not imply that cultural context has no pedagogical significance. Rather, it suggests that the categorical labels used in the dataset did not explain the observed variation. A nominal category such as Indigenous ethnoscience may include interventions ranging from superficial use of cultural examples to sustained engagement with community knowledge holders, local language, epistemic comparison, and culturally governed knowledge practices. Conversely, an intervention coded as local context may integrate community knowledge more deeply than one labelled ethnoscience. Research on science–Indigenous knowledge integration has consistently emphasized that implementation depends on teachers’ epistemological positions, practical reasoning, and capacity to mediate between knowledge systems ( Ogunniyi, 2007). More recent studies also report that teachers encounter challenges related to curriculum alignment, resource availability, confidence, and the design of culturally appropriate practical work ( Mdleleni & Ngcoza, 2025; Mlotshwa & Tsakeni, 2024). Evidence from Lombok further indicates that local content must be made explicit in primary science teachers’ lesson planning, reinforcing the importance of operationalizing culturally relevant knowledge at the curriculum-planning stage ( Ali et al., 2026). Future moderator coding should therefore assess the depth and quality of cultural integration rather than relying only on broad contextual labels ( Anggreini, 2024; Fitria, 2025).

Media type was the only moderator with a statistically significant omnibus test, but this result should remain exploratory. Several categories were represented by very few studies, and the estimate for digital platforms and open educational resources was accompanied by an extremely wide confidence interval that crossed zero. More importantly, the media categories were not fully equivalent at the conceptual level. AI tools, simulations, modules, digital platforms, and games can reasonably be treated as instructional resources, whereas conceptual curriculum frameworks and teacher professional-development programmes describe broader intervention formats, and a survey questionnaire is primarily a measurement instrument rather than a teaching medium ( Putra et al., 2025). The significant omnibus result may therefore partly reflect category construction rather than a stable difference in the effectiveness of comparable media. The data indicate variation across the coded categories, but they do not justify ranking technologies or concluding that one medium is inherently superior. The contribution of media is likely conditional on instructional design, accessibility, teacher mediation, cultural authenticity, and alignment with the assessed outcome.

Intervention duration also did not explain a statistically detectable proportion of the variation. Medium and long-or-non-intervention categories produced somewhat larger point estimates than short interventions, but the omnibus test was nonsignificant. The combined category “long or non-intervention” limits substantive interpretation because an extended intervention and a study without a defined intervention duration are not comparable conditions. Duration should ideally be coded as the number of weeks, sessions, instructional hours, or total exposure time, with non-intervention studies coded as not applicable. A continuous or more consistently defined duration variable would permit a more defensible examination of possible dose–response patterns.

The funnel plot showed visible asymmetry, including a small number of imprecise studies with large positive effects. This pattern is compatible with possible small-study effects, but it is insufficient to confirm publication bias. Funnel-plot asymmetry may also result from genuine heterogeneity, differences in methodological quality, selective outcome reporting, or an association between study size and intervention characteristics. Given the high I ^2^ value, heterogeneity is a plausible contributor to the observed pattern. A formal regression-based asymmetry test, influence diagnostics, and sensitivity analyses excluding extreme effects would provide a stronger basis for evaluating the robustness of the pooled estimate. Even if statistical asymmetry were detected, it should be described as evidence of small-study effects rather than treated automatically as proof of selective publication.

The findings have several implications for theory and practice. Theoretically, they support an account in which cultural knowledge functions as an epistemic and pedagogical resource rather than as decorative content. Contextualized science instruction may reduce the distance between students’ lived experience and school science, but its value depends on whether students are enabled to investigate, compare, justify, and critically examine explanations. Effective integration should preserve the integrity and contextual meaning of local or Indigenous knowledge while also making the evidentiary practices of science explicit. This requires more than replacing textbook examples with local objects; it involves designing tasks in which cultural practices generate questions, data, models, argumentation, and reflection. For practice, the results support cautious adoption of culturally grounded science and STEM instruction, particularly when it is embedded in structured inquiry, collaborative investigation, or project work. Teachers need support in identifying culturally relevant phenomena, engaging respectfully with communities, and avoiding the reduction of Indigenous knowledge to decontextualized facts. Professional learning should address both pedagogical design and epistemological mediation. Recent research indicates that teachers’ ability to integrate Indigenous knowledge is shaped by their topic-specific pedagogical content knowledge, confidence, access to culturally appropriate resources, and opportunities to work with knowledgeable community members. Technology may support representation, simulation, and access to resources, but it should be selected for its pedagogical function rather than for novelty.

Several limitations constrain the strength of the conclusions. First, the very high heterogeneity limits the generalizability of the pooled mean. Second, some subgroups were small and imprecise, reducing the power of moderator tests. Third, several coding categories combined conceptually different units of analysis, particularly media type, curriculum status, and intervention duration. Fourth, standardized mean differences permit numerical synthesis across instruments but do not eliminate differences in construct validity, reliability, or sensitivity ( Asamoah et al., 2024; Zigon, 2023). Fifth, the funnel plot suggests possible small-study effects, although their source remains uncertain. Sixth, the use of aggregate study-level data prevents examination of individual-level characteristics such as prior achievement, language, socioeconomic background, and degree of cultural identification. Finally, the interpretation assumes that all included estimates are independent and statistically compatible; the analytical dataset should therefore be checked carefully to ensure that duplicate, one-group, conceptual, or non-intervention records have not been pooled as if they were equivalent controlled comparisons.

Future research should use stronger controlled designs, report baseline comparability and attrition transparently, and provide complete descriptive statistics for all groups and time points. Studies should document intervention fidelity and describe cultural integration in operational terms, including community involvement, use of local language, the role of knowledge holders, ethical governance of Indigenous knowledge, and the way different explanatory systems are compared. Future meta-analyses should separate controlled, one-group, and non-intervention designs; use multilevel or robust-variance models when studies contribute dependent outcomes; report prediction intervals; and restrict multivariable meta-regression to theoretically justified moderators with adequate representation. These steps would help distinguish whether observed effects arise from cultural relevance itself, from associated active-learning strategies, or from the interaction between the two.

## 5. Conclusion

This updated systematic review and meta-analysis indicates that the examined ethnoscience-, indigenous knowledge-, local wisdom-, and culturally contextualized approaches were associated, on average, with more favourable science and STEM educational outcomes than the comparison conditions. The pooled standardized mean difference was positive and comparatively large (g = 1.17, 95% CI [0.88, 1.46]). However, the substantial heterogeneity (I ^2^ = 91.5%) and the wide range of study-level estimates show that the magnitude of the association was not consistent across studies. The pooled value should therefore be interpreted as an average across diverse interventions and settings, not as an effect that can be expected uniformly in every educational context. Most prespecified moderators, including educational level, outcome type, instructional model, science subject, cultural context, and intervention duration, did not show statistically detectable between-category differences. Media type was the only moderator with a statistically significant omnibus test, but the result was based partly on small, imprecise, and conceptually heterogeneous subgroups. It consequently provides evidence of possible variation across the coded categories rather than a basis for ranking particular media or technologies. The funnel plot also suggested possible small-study effects, although the observed asymmetry cannot be attributed exclusively to publication bias because the evidence base was highly heterogeneous.

The findings support a conditional interpretation of culturally grounded science education. Such approaches may be educationally valuable when cultural knowledge is integrated authentically, connected explicitly to scientific concepts and evidence, and enacted through coherent inquiry, collaboration, projects, or other appropriately scaffolded pedagogies. Their contribution is likely to depend on implementation quality, teacher mediation, community engagement, measurement quality, and the alignment between intervention activities and assessed outcomes. Future research should employ stronger controlled designs, report complete statistical information, distinguish clearly among study designs, document the depth of cultural integration and implementation fidelity, and provide sufficient data for prediction intervals and more stable moderator analyses. Overall, the synthesis provides qualified support for the use of ethnoscience and culturally contextualized approaches in science and STEM education, while also demonstrating that the current evidence does not justify a universal causal claim. The most defensible conclusion is that these approaches are associated with positive average outcomes under the conditions represented in the available studies, but the size and consistency of those outcomes remain context-dependent and require further methodologically rigorous investigation.

## Author contributions

Muhammad Fajar Marsuki and Sony Yunior Erlangga conceived and supervised the study, coordinated the review protocol, and led manuscript preparation. Lalu Usman Ali contributed to conceptual development, search design, interpretation, and critical revision. Ratih Permana Sari, Nur Azizah Lubis, and Florentina Maria Panda conducted literature searching, screening, data extraction, risk-of-bias assessment, moderator coding, data verification, reference management, and preparation of tables and figures. Septi Aprilia and Dimas Fahrudin performed and validated the effect-size calculations, meta-analysis, moderator and sensitivity analyses, and visualizations. All authors interpreted the findings, reviewed and approved the final manuscript, and accepted accountability for the integrity of the work.

## Data Availability

Zenodo: Supporting Data and Reporting Materials for “Educational Effects of Ethnoscience, Indigenous Knowledge, and Local Wisdom in Science and STEM Education”. https://doi.org/10.5281/zenodo.22226433 ( Marsuki et al., 2026). The repository contains study-level quantitative data supporting the systematic review and meta-analysis, including sample sizes, means, standard deviations, moderator coding, and analytical information used to generate the reported effect-size estimates and meta-analytic results. Zenodo: Supporting Data and Reporting Materials for “Educational Effects of Ethnoscience, Indigenous Knowledge, and Local Wisdom in Science and STEM Education”. https://doi.org/10.5281/zenodo.22226433 ( Marsuki et al., 2026). The repository contains the completed PRISMA 2020 checklist, PRISMA 2020 flow diagram, search strategy and study-selection documentation, data-extraction and moderator-coding materials, methodological quality and risk-of-bias documentation, and supporting analytical materials. The underlying data and extended data are available under the Creative Commons Zero (CC0 1.0) Public Domain Dedication. The completed PRISMA 2020 Checklist and PRISMA 2020 Flow Diagram are available in the Zenodo repository at https://doi.org/10.5281/zenodo.22226433 ( Marsuki et al., 2026) under a CC0 1.0 license.
