# Supplementary material for: Educational Effects of Ethnoscience, Indigenous Knowledge, and Local Wisdom in Science and STEM Education
Source: F1000Res. 2026 Sep 22;15:1565. [Version 1] doi: 10.12688/f1000research.189428.1 (PMC13691352; doi:10.12688/f1000research.189428.1)
Supplement: Peer review history

# Reviewer report: Reviewer response for version 1

**DOI:** 10.5256/f1000research.209068.r525380

**Published:** 2026-10-22

**Authors:** Rizky Agassy Sihombing

**Affiliations:** 1 National Taiwan Normal University, Taipei, Taiwan

**General assessment**

 This manuscript addresses a relevant question in science and STEM education by synthesizing quantitative evidence on ethnoscience, Indigenous knowledge, local wisdom, and culturally contextualized instruction. The use of a systematic review and random-effects meta-analysis is potentially valuable, particularly because the authors examine educational outcomes across different populations, instructional approaches, and cultural contexts. The manuscript also appropriately acknowledges the substantial heterogeneity among the included studies and avoids interpreting the pooled estimate as a uniform effect.

 However, several methodological and reporting issues need clarification before the quantitative findings can be considered sufficiently reliable. The most important concerns involve the consistency between the eligibility criteria and the study designs represented in the synthesis, the operationalization of moderator categories, and the accuracy and completeness of statistical reporting. I recommend major revision, with particular attention to the following points.

 Major comments

**1. Consistency between eligibility criteria and included study designs**

 The eligibility criteria specify experimental, quasi-experimental, or controlled comparative studies with an intervention and a comparison condition. However, the coding framework includes a one-group pretest–posttest design, while the results refer to curriculum-conceptual studies and a “long-or-non-intervention” duration category.

 The authors should clarify whether these records were eligible under the original protocol and whether they contributed to the pooled estimate. If studies without a valid comparison condition or without an actual intervention were included, the authors should explain and justify this decision. Where appropriate, the analyses should distinguish controlled comparisons from one-group designs and other methodologically different records, with sensitivity analyses to assess their influence on the overall estimate.

**2. Clarification and consistency of moderator coding**

 The moderator categories appear to include conceptually different constructs. For example, the media-type analysis includes instructional resources, professional-development programmes, curriculum frameworks, and survey-questionnaire categories. Similarly, the duration analysis combines long interventions with non-intervention studies.

 These categories are not directly equivalent and may complicate the interpretation of moderator effects. The authors should provide a complete study-level coding table, explain how each category was assigned, and clarify whether the reported categories match the prespecified coding framework. Categories representing different constructs should be separated or reclassified where possible. The authors should also report the research-design moderator that is described in the Methods section.

**3. Verification of statistical reporting**

 Several reported confidence intervals appear internally inconsistent and should be checked against the original statistical output. For example, the estimate for Londoño-Proaño and Pérez-Gavilanes is reported as (g=0.03), with a confidence interval of [0.31, 0.24]. Other subgroup intervals, including those for motivation, awareness, and attitudes; digital platforms and open educational resources; and game- and narrative-based media, also warrant verification.

 I do not assume that the underlying analyses are necessarily incorrect. However, the authors should verify the point estimates, confidence limits, subgroup sample sizes, and associated p-values against the analysis dataset and correct any transcription or reporting errors. The forest plot, subgroup tables, and narrative descriptions should be fully consistent.

**4. Robustness of the pooled effect**

 The pooled estimate is positive and relatively large ((g=1.17)), but heterogeneity is substantial ((I^2=91.5%)). The manuscript also reports an unusually large individual effect ((g=4.74)). Such an estimate may be valid, but its influence on the pooled result should be examined.

 The authors should report appropriate influence and sensitivity analyses, including the effect of excluding the most extreme study and, where feasible, studies judged to have a high risk of bias. A prediction interval would also help readers understand the range of effects that might plausibly occur in comparable future settings. These analyses would clarify how robust the main conclusion is to influential studies and methodological differences.

**5. Search strategy and reproducibility**

 The manuscript provides a general search structure and describes several supplementary search methods. Nevertheless, reproducibility would be improved by reporting the exact names of all databases and registers, the search date for each source, and the complete search strings as executed in each database. The authors should also ensure that the PRISMA flow diagram and the narrative description consistently account for identified records, duplicates, records removed before screening, reports sought for retrieval, and studies included.

 The statement that a protocol was developed but not externally registered is appropriately transparent. The authors should retain this information and clarify whether the final analysis and coding procedures followed the prespecified protocol.

 Additional comments

1. Please provide a clearer account of the risk-of-bias findings and the results of the sensitivity analyses mentioned in the Methods section.
2. The significant omnibus result for media type should remain exploratory. The authors should avoid interpreting it as evidence that a specific medium is superior, given the small subgroups and conceptual differences among categories.
3. The funnel plot should be interpreted cautiously. If a formal asymmetry test is conducted, its result and limitations should be reported; otherwise, the current interpretation should remain descriptive.
4. Please ensure that the tables, figures, and text use consistent terminology for moderator categories and provide sufficient information to understand the number of studies contributing to each analysis.
5. The conclusion should continue to distinguish the positive average association from a uniform or universally generalizable intervention effect.

**Overall recommendation**

 The manuscript addresses an important topic and has the potential to contribute to the literature on culturally contextualized science and STEM education. The main concerns are potentially resolvable through clarification of study eligibility, consistent moderator coding, verification of statistical reporting, and additional robustness checks. I therefore recommend **major revision** rather than rejection, provided that the authors can demonstrate that the included studies and reported estimates are appropriate for the stated meta-analytic design.

Are the rationale for, and objectives of, the Systematic Review clearly stated?

Yes

Is the statistical analysis and its interpretation appropriate?

Partly

If this is a Living Systematic Review, is the ‘living’ method appropriate and is the search schedule clearly defined and justified? (‘Living Systematic Review’ or a variation of this term should be included in the title.)

Partly

Are sufficient details of the methods and analysis provided to allow replication by others?

Partly

Are the conclusions drawn adequately supported by the results presented in the review?

Yes

Reviewer Expertise:

Systematic Literature Review, ESD, Scientific Literacy, and Science Education

I confirm that I have read this submission and believe that I have an appropriate level of expertise to confirm that it is of an acceptable scientific standard, however I have significant reservations, as outlined above.

# Reviewer report: Reviewer response for version 1

**DOI:** 10.5256/f1000research.209068.r525370

**Published:** 2026-10-13

**Authors:** Andika Febrian

**Affiliations:** 1 Universitas Sultan Ageng Tirtayasa, Serang, Indonesia

**1. Clarity of Terminology**: The author needs to explain the conceptual differences between Indigenous Knowledge, Local Wisdom and Ethnoscience, covering their characteristics, theoretical foundations and contexts of use. All three have different scopes and implications and therefore cannot simply be used interchangeably. This explanation is necessary to clarify the conceptual framework and avoid terminological ambiguity, as not all readers will be familiar with the distinctions between these three terms.

**2. Geographical and Contextual Scope**: Authors should clarify the geographical scope of the study and the cultural context under review, specifying whether it is limited to a single country, covers a regional or continental area, or is global in scale.

**3. Database Transparency**: Authors must explicitly state the names of all databases (Scopus/WoS/etc.) used to identify articles, including any additional search sources where applicable. This information is essential to ensure the transparency and reproducibility of the literature search.

**4. Completeness of PRISMA reporting**: PRISMA reporting must include specific search strategies for each database, search date ranges, and study screening procedures.

**5. Table of Study Characteristics**: The authors should present a table summarising the characteristics of all 31 studies, including authors/year, sample, study design, intervention (if any), and main findings.

Are the rationale for, and objectives of, the Systematic Review clearly stated?

Yes

Is the statistical analysis and its interpretation appropriate?

Yes

If this is a Living Systematic Review, is the ‘living’ method appropriate and is the search schedule clearly defined and justified? (‘Living Systematic Review’ or a variation of this term should be included in the title.)

Yes

Are sufficient details of the methods and analysis provided to allow replication by others?

Yes

Are the conclusions drawn adequately supported by the results presented in the review?

Yes

Reviewer Expertise:

Physics Education, Science Education, Ethnoscience; Indigenous Knowledge; Local Wisdom; Local Potencials in Science Learning

I confirm that I have read this submission and believe that I have an appropriate level of expertise to confirm that it is of an acceptable scientific standard.

# Reviewer report: Reviewer response for version 1

**DOI:** 10.5256/f1000research.209068.r525378

**Published:** 2026-09-30

**Authors:** Gladys Sunzuma

**Affiliations:** 1 Bindura University of Science Education, Bindura, Mashonaland Central Province, Zimbabwe

The methodology should include the names of the databases. 

 The PRISMA methodology is insufficiently reported - complete database-specific search strings, search dates and screening procedures should be provided.

 There is no detailed characteristics table for all 31 studies - a systematic review should clearly report author/year, sample, design, intervention, etc,.

Are the rationale for, and objectives of, the Systematic Review clearly stated?

Yes

Is the statistical analysis and its interpretation appropriate?

Yes

If this is a Living Systematic Review, is the ‘living’ method appropriate and is the search schedule clearly defined and justified? (‘Living Systematic Review’ or a variation of this term should be included in the title.)

Yes

Are sufficient details of the methods and analysis provided to allow replication by others?

Partly

Are the conclusions drawn adequately supported by the results presented in the review?

Yes

Reviewer Expertise:

Mathematics education

I confirm that I have read this submission and believe that I have an appropriate level of expertise to confirm that it is of an acceptable scientific standard, however I have significant reservations, as outlined above.
